# Infection with the Endonuclear Symbiotic Bacterium *Holospora obtusa* Reversibly Alters Surface Antigen Expression of the Host *Paramecium caudatum*

**DOI:** 10.3390/microorganisms13050991

**Published:** 2025-04-25

**Authors:** Masahiro Fujishima

**Affiliations:** Department of Environmental Science and Engineering, Graduate School of Science and Engineering, Yamaguchi University, Yoshida 1677-1, Yamaguchi 753-8512, Japan; fujishim@yamaguchi-u.ac.jp

**Keywords:** *Paramecium caudatum*, *Holospora obtusa*, endonuclear symbiotic bacteria, bacterial infection, endosymbiosis, phenotypic change in the host, surface antigen, immobilization antigen, GPI-anchored surface protein

## Abstract

It is known that the ciliate *Paramecium* cell surface including cilia is completely covered by high-molecular-mass GPI-anchored proteins named surface antigens (SAgs). However, their functions are not well understood. It was found that ciliate *Paramecium caudatum* reversibly changes its SAgs depending on the absence or presence of the endonuclear symbiotic bacterium *Holospora obtusa* in the macronucleus. Immunofluorescence microscopy with a monoclonal antibody produced SAg of the *H. obtusa*-free *P. caudatum* strain RB-1-labeled cell surface of the *H. obtusa*-free *P. caudatum* RB-1 cell but not the *H. obtusa*-bearing RB-1 cell. When this antibody was added to the living *P. caudatum* RB-1 cells, only *H. obtusa*-free cells were immobilized. An immunoblot with SAgs extracted from *Paramecium* via cold salt/ethanol treatment showed approximately 266-kDa SAgs in the extract from *H. obtusa*-free cells and 188 and 149-kDa SAgs in the extract from *H. obtusa*-bearing cells. *H. obtusa*-free RB-1 cells produced from *H. obtusa*-bearing cells via treatment with penicillin-G-potassium re-expressed 266-kDa SAg, while the 188 and 149-kDa SAgs disappeared. This phenotypic change in the SAgs was not induced by degrees of starvation or temperature shifts. These results definitively show that *Paramecium* SAgs have functions related to bacterial infection.

## 1. Introduction

The surface of the cell body and the cilia of the ciliate *Paramecium* is covered by a 20–30 nm thick layer of high-molecular-weight proteins of 250–300 kDa [1,2,3,4,5,6,7]. In *Paramecium,* this protein accounts for about 30% of the ciliary proteins and is named the surface antigen (SAg) or immobilization antigen (i-antigen) because the protein is exposed outside the cell surface and the swimming behavior of *Paramecium* cells incubated with antibodies against the SAg is immobilized [8,9,10]. *Paramecium* SAg is a glycosylphosphatidylinositol (GPI)-anchored protein with a cysteine periodicity in the entire SAg [11]. It is tethered to the cell surface through a lipid anchor [12,13,14]. *Paramecium* strains with specific SAgs have been called serotypes. Recently, the genomes of six different *Paramecium* species were screened for serotype genes and allowed the identification of the subfamilies of the isogenes of individual serotypes that were mostly represented by intrachromosomal gene duplicates [15].

In other organisms, these SAgs fulfill a variety of functions, including the transport of folate [16,17], channel activation [18], and the binding of ligands for signal transduction [19,20,21]. It has been established that the loss of GPI anchoring affects morphogenesis and sporulation in yeast [22] and that GPI-anchored proteins are essential for the growth of *Trypanosoma brucei* but not for *Leishmania mexicana* in mammalian cells [23,24]. Furthermore, defects in proteins essential for the localization of GPI-anchored proteins to the cell membrane surface in mice (PIG-A knockout mice) do not survive birth [25], and GPI anchoring is necessary for proper skin differentiation and maintenance [26]. SAg has also been studied in the ciliate *Tetrahymena thermophila*, but its function remains unknown [27]. However, despite the large number of published articles on SAgs in *Paramecium* over the past 100 years, their functions remain poorly understood. Yano et al. [28] and Paquette et al. [17] definitively showed specific defects in the chemoresponse to glutamate and folate when the synthesis of GPI-anchored intermediates was reduced, while growth, mating, and swimming behavior seemed to be normal. Conversely, it has been documented that the expressed *Paramecium* SAg molecules exhibit a response to variations in temperature, pH, salt concentration, type of culture medium, feeding rate, antisera, proteolytic enzymes, UV and X-ray irradiation, patulin, and other antibiotics [29]. Therefore, *Paramecium* SAg is considered to play a role in adapting to changes in various environmental factors in the field.

The present study is concerned with the phenotypic changes of *P. caudatum* cells following successful endosymbiosis with endonuclear symbiotic bacteria of the *Holospora* species to the host nucleus. As previously demonstrated, the infection of endonuclear symbiotic bacteria of the *Holospora* species results in alterations in the gene expressions of the host concerning intracellular signaling, transcription, and aerobic metabolism [30], as well as heat shock proteins [31,32]. The objective of this study is to determine whether endosymbiosis with the macronucleus-specific *H. obtusa* modifies the host *P. caudatum* SAgs and whether the changes in the SAgs are reversible or irreversible, depending on the infection of *H. obtusa*. Additionally, this study will explore whether the changes in SAgs occur in the same SAgs as those induced by other factors or the specific SAg for infection of *H. obtusa.*

## 2. Materials and Methods

### 2.1. Strains and Cultures

The *Holospora obtusa*-bearing (symbiotic) *Paramecium caudatum* strain RB-1 (syngen 4, mating type E) infected with *H. obtusa* strain F1 and *H. obtusa*-free (aposymbiotic) RB-1 cells was mainly used in this study. Strain RB-1 cells were collected in 1993 in Stuttgart, Germany by Hans-Dieter Görtz. *H. obtusa* strain F1 cells were isolated by M. Fujishima using Percoll P4937 (Merk, Darmstadt, Germany) density gradient centrifugation [33] from symbiotic *P. caudatum* strain c103 collected by H.-D. Görtz (syngen and collected year and place, unknown), and infected the aposymbiotic *P. caudatum* strain RB-1 cell by mixing them. Symbiotic and aposymbiotic paramecia were cultivated in glass test tubes (18 mm × 180 mm) with a modified Dryl’s solution (MDS) (KH_2_PO_4_ was used instead of NaH_2_PO_4_·2H_2_O) [33,34] containing 1.25% (*w*/*v*) fresh lettuce juice and 0.0001% (*w*/*v*) stigmasterol (Tama Biochemical Co., Ltd., Tokyo, Japan) at 25 °C. The MDS containing fresh lettuce juice and stigmasterol was inoculated with a non-pathogenic *Klebsiella pneumoniae* strain 6081 one day before use at 25 °C [35]. Strain RB-1 of *P. caudatum*, 14 strains of *P. aurelia* species, and 1 strain each of *P. polycarium*, *P. jenningsi*, *P. duboscqui*, *P. calkinsi,* and *P. multimicronucleatum* were also used. The strain names of these *Paramecium* species are given in Table 1 of the results section. The strains of *Paramecium* used were those of preserved strains in the Fujishima laboratory at Yamaguchi University, Japan. Subsequently, all strains utilized were deposited into the National BioResource Project *Paramecium* (NBRP-*Paramecium*, http://nbrpcms.nig.ac.jp/paramecium/) established by M. Fujishima in 2012 at Yamaguchi University (accessed on 30 November 2021).

### 2.2. Extraction of P. caudatum Surface Antigens by Cold Salt/Ethanol Ttreatment

The surface antigens (SAgs) for aposymbiotic and symbiotic *P. caudatum* cells were extracted according to the procedure described by Preer [10]. One liter of *Paramecium* culture in the stationary phase of growth (about 1.0 × 10^6^ cells) was centrifuged at 150 g for 3 min and resuspended in 200 μL of MDS. Then, 800 μL of cold salt/ethanol (10 mM Na_2_HPO_4_, 150 mM NaCl, 30% [*v*/*v*] ethanol) was added, and the mixture was kept on ice for 1 h. The cell suspension was centrifuged at 25,000× *g* for 3 min at 4 °C and the supernatant was harvested and mixed with 150 μL of 0.2N HCl with stirring to reduce its pH to 2.0. Then, the supernatant was centrifuged by the same centrifugation, and the pH of the supernatant was increased to 7.0 by adding an equal volume of 0.2N NaOH. The total volume of the extracts was concentrated to 100 μL by ultrafiltration (Sartorius, Göttingen, Germany).

### 2.3. Production of Monoclonal Antibody

A monoclonal antibody (mAb) specific to the SAg of aposymbiotic *P. caudatum* strain RB-1 was developed using the method of Galfre and Milstein [36]. Aposymbiotic *P. caudatum* strain RB-1 cells (2 × 10^4^ cells) suspended in phosphate-buffered saline (PBS, 137 mM NaCl, 2.68 mM KCl, 8.1 mM NaHPO_4_·12H_2_O, 1.47 mM KH_2_PO_4_, pH 7.2) were injected into the peritoneal cavity of BALB/c mice. The first injection was followed by two booster injections at 2-week intervals. The hybridoma cells producing mAb against the SAg of aposymbiotic *P. caudatum* RB-1 cells were screened by indirect immunofluorescence microscopy with fluorescein isothiocyanate (FITC)-conjugated goat anti-mouse IgG (G0406, Tokyo Chemical Industry Co., Ltd., Tokyo, Japan) and cloned by limiting dilution [37]. This mAb was named mAb SAgPC and used for indirect immunofluorescence microscopy and immunoblotting in this study. For the development of another mAb specific to the groEL homolog of *H. obtusa* strain F1, according to Iwatani et al. [38], extracts from excised spots of the *H. obtusa* groEL homolog on two-dimensional sodium dodecyl sulfate-polyacrylamide gel electrophoresis (2D-SDS-PAGE) gels stained with Coomassie brilliant blue R250 (CBB) (Merck, Darmstadt, Germany) were used as an antigen. This mAb was confirmed not to cross-react with *P. caudatum* nor its prey, *K. pnoemoniae* strain 6081, by indirect immunofluorescence microscopy, and used to confirm that *H. obtusa* in the host *P. caudatum* RB-1 cells was completely eliminated by penicillin treatment.

### 2.4. Indirect-Immunofluorescence Microscopy

*Paramecium* cells were washed with PBS, dried on micro-cover grasses (4.5 × 24 mm, 0.12–0.17 µm thickness, Matsunami micro-cover glass (Kishiwada, Oosaka, Japan), fixed with 4% (*w*/*v*) paraformaldehyde for 5 min on ice, washed twice with PBS for 10 min each, and incubated with mAb SAgPC for 1 h at 25 °C. The cover glasses were washed twice with PBS for 10 min each at 4 °C and incubated with fluorescein isothiocyanate (FITC)-conjugated goat anti-mouse IgG (Molecular Probes, Eugene, OR, USA) for 1 h at 25 °C. Then, the cover glasses were washed twice with PBS for 10 min each at 4 °C and observed with a fluorescence microscope, Olympus BH2-RFL (Tatsuno, Nagano, Japan) using the DPlanApo40UV objective. To label live *Paramecium* cells with mAb SAgPC, cell suspensions of 250 μL of cells in the early stationary phase of growth were mixed with 50 μL of hybridoma culture medium containing mAb SAgPC for 30 min at 25 °C, washed twice with MDS, mixed with 50 μL of FITC-conjugated goat anti-mouse IgG for 30 min at 25 °C, and observed using a fluorescence microscope.

### 2.5. SDS-PAGE and Immunoblotting

The cold salt/ethanol extracts were mixed with an equal volume of Laemmli’s lysis buffer [39] and boiled for 5 min. Then, 25 μL each of the samples obtained from aposymbiotic and symbiotic cells was subjected to 5% (*w*/*v*) SDS-PAGE. The gels were stained with CBB or electroblotted onto the Immobilon-P Poly Vinylidene DiFluoride (PVDF) membrane (Millipore, Bedford, MA, USA). The membrane was incubated with mAb SAgPC for 6 h at room temperature, and the SAg band was detected using Goat anti-mouse IgG + IgM, Alkaline Phosphatase (Biosource, Camarillo, CA, USA), and the BCIP/NBT phosphatase substrate (KPL, Gaithersburg, MD, USA). 2D-SDS-PAGE was performed as previously described by Iwatani et al. [38]. Pre-stain molecular mass markers (Nacalai Tesque Inc., Kyoto, Japan) were used.

### 2.6. Immobilization Test

The methodology for the SAg immobilization test followed that of previous papers [9,40]. Briefly, a cell suspension of 250 μL of aposymbiotic cells in the early stationary phase of growth was mixed with 50 μL of hybridoma culture medium containing mAb SAgPC at a density of 5000 cells/mL at 25 °C, and we observed whether the cells were immobilized or not every 5 min for the first 30 min, every 10 min for 30–60 min, at 90 min, and every 60 min for 90–360 min. As a control experiment, the cell suspension was mixed with 50 μL of MDS.

Four cover glasses of 4.5 × 24 mm (Matsunami micro-cover glass, Japan) were placed on a glass slide to form a rectangular container for the cell culture medium. An aliquot of cell suspension with or without the mAb (150 µL) was transferred to the container and covered with a 24 × 50 mm cover glass. The glass slide was placed on the stage of a stereomicroscope Olympus SZ-61(Tatsuno, Nagano, Japan) 270 min after mixing with the mAb at 25 °C. Photomicrographs of the swimming loci of the cells were taken under dark-field illumination with an exposure time of 2 s using Fuji chrome 1600 film (Fuji-Film, Tokyo, Japan).

### 2.7. Creation of Aposymbiotic Cells from Symbiotic Cells

In order to remove *H. obtusa* from the macronucleus of symbiotic *P. caudatum* strain RB-1 cells, paramecia were cultivated in a culture medium containing penicillin-G-potassium (ICN, Cincinnati, OH, USA). A stock solution of penicillin-G-potassium was prepared as follows: the antibiotic was dissolved in 10 mM Na-PB, pH 7.0 at a concentration of 25,000 units/mL and kept at −30 °C until use. A quantity of 4.9 mL of the culture medium containing symbiotic cells was mixed with 0.1 mL of a penicillin-G-potassium solution, resulting in an overall concentration of 250 units/mL of penicillin [41]. Then, 5 mL of the fresh culture medium was added on successive days to dilute the antibiotics. Paramecia were observed under a differential-interference-contrast microscope (DIC) and indirect immunofluorescence microscopy on an Olympus BH2-RFL using DPlanApo40UV and DPlanApo100UVPL (Olympus, Tatsuno, Nagano, Japan) with anti-*H. obtusa* groEL mAb to confirm the absence of *H. obtusa* in the host macronucleus. Three days after the penicillin treatment, most of *P. caudatum* strain RB-1 cells were free of *H. obtusa*, and an aposymbiotic RB-1 clone was established by cloning the paramecia.

### 2.8. Starvation and Temperature-Shift Stress

Aposymbiotic *P. caudatum* RB-1 cells (2 × 10⁵ cells) in the early stationary phase of growth were transferred to a centrifuge tube equipped with a 15 µm pore nylon mesh and then filtered. A volume of 100 mL of MDS was added to the cells, which were then washed three times and suspended in 500 mL of sterile MDS. Five milliliters of the cell suspension were used for indirect immunofluorescence microscopy, while the remaining 495 mL was used for the extraction of SAgs via the cold salt/ethanol extraction method [10]. Indirect immunofluorescence microscopy was conducted 0, 1, 2, 4, 7, and 14 days following the induction of starvation, with 150–200 cells being observed in each instance. The extraction of SAgs was performed 0, 2, 4, and 7 days after the initiation of starvation, with the extracted SAgs being utilized for SDS-PAGE and immunoblotting with mAb SAgPC.

In order to examine the effects of different temperatures on the expression of SAg in aposymbiotic *P. caudatum* RB-1 cells, the cells were cultivated in 500 mL of the culture medium at 25 °C. Approximately 2 × 10^5^ cells in the early stationary phase of growth were transferred to 10, 15, 25, and 35 °C for 24 h. Thereafter, their SAgs were extracted using the cold salt/ethanol extraction method. The types of SAgs expressed in the cells were then examined via SDS-PAGE and immunoblotting with mAb SAgPC.

## 3. Results

### 3.1. SAgs Comparison Between Symbiotic and Aposymbiotic Paramecium Cells

**Figure 1:**
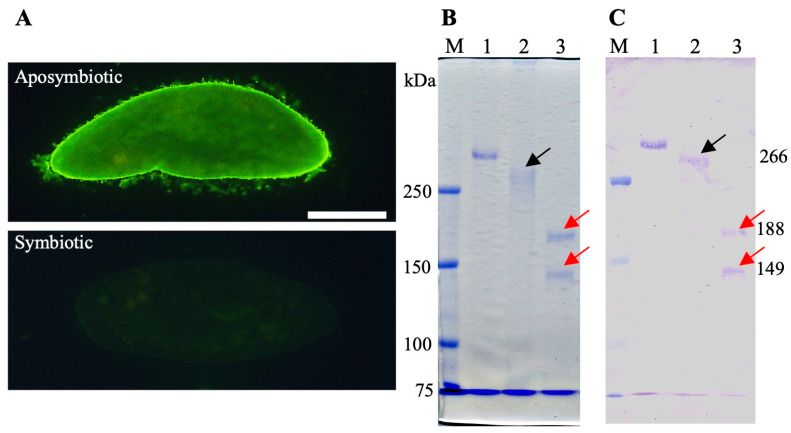
Indirect immunofluorescence and immunoblot of aposymbiotic and symbiotic *P. caudatum* RB-1 cells with mAb SAgPC. *Paramecium* cells in the early stationary phase of growth were used. This mAb was developed by injecting whole cells of the aposymbiotic *P. caudatum* RB-1 cells into mice. (**A**) Indirect immunofluorescence micrographs of aposymbiotic and symbiotic RB-1 cell. Note that the cilia and cell body show strong FITC fluorescence in the aposymbiotic cell but not in the symbiotic cell infected with *H. obtusa* F1 in the macronucleus. (**B**) CBB-stained SDS-PAGE gel of cold salt/ethanol extracts. (**C**) Immunoblot with mAb SAgPC. Lane 1, extracts of *P. tetraurelia* stock 51 cells. Lane 2, extract of aposymbiotic *P. caudatum* RB-1 cells. Lane 3, extract of symbiotic *P. caudatum* RB-1 cells. Lane M, pre-stained molecular mass markers. Note that mAb cross-reacted with a high molecular mass SAg of *P. tetraurelia* stock 51. Black arrow, aposymbiotic *P. caudatum* RB-1-specific SAg (about 266-kDa). Red arrow, symbiotic *P. caudatum* RB-1-specific SAgs (188 and 149-kDa). Since the molecular masses of the SAgs of *P. tetraurelia* strain 51 are reported to be from 250 to 300 kDa [4], using the band in lane 1 as a 300-kDa marker, the molecular mass of a band extracted from aposymbiotic *P. caudatum* RB-1 cells was calculated to be 266-kDa (lane 2). Note that even though two low-molecular-weight SAg bands were detected by immunoblotting in symbiotic *P. caudatum* (**B**,**C**), FITC fluorescence was only detected in symbiotic *P. caudatum* by indirect immunofluorescence microscopy (**A**). Scale bar, 50 μm.

A monoclonal antibody (mAb) SAgPC specific to the SAg of the aposymbiotic *P. caudatum* RB-1 cell was developed by injecting aposymbiotic *P. caudatum* RB-1 cells into mice. As demonstrated in Figure 1A, indirect immunofluorescence microscopy using the mAb revealed strong FITC fluorescence on the cell surface of the aposymbiotic RB-1 cell, including its cilia. In contrast, the symbiotic RB-1 cell did not exhibit fluorescence. This phenomenon was induced in symbiotic RB-1 within 20 days of infection with *H. obtusa*. Furthermore, living aposymbiotic RB-1 cells also exhibited FITC fluorescence on their surface, while living symbiotic cells did not. This phenomenon indicates that the epitope in the SAg is reactive to the mAb and exposed outside the cell surface in the living aposymbiotic *P. caudatum* RB-1 cell, but not in the living symbiotic RB-1 cell. However, as shown in Figure 1B,C, cell extracts obtained from *P. tetraurelia* stock 51 (lane 1), aposymbiotic *P. caudatum* RB-1 (lane 2), and symbiotic *P. caudatum* RB-1 (lane 3) via cold salt/ethanol extraction revealed the presence of bands labeled with the mAb SAgPC. This extraction medium has been widely used for the extraction of SAg proteins from various *Paramecium* species [10]. In order to ascertain the efficacy of the SAg extraction method for *Paramecium* cells, a cell extract of *P. tetraurelia* strain 51 was also utilized. This strain is among the most frequently employed for *Paramecium* SAg experiments. Consequently, a high-molecular-mass band that had been stained with CBB was detected in the extract from the stock 51 cells (Figure 1B, lane 1), though the stock 51 cells did not show FITC fluorescence via indirect immunofluorescence microscopy. It is well established that the molecular masses of the SAgs of *P. tetraurelia* stock 51 range from 250 kDa to 300-kDa [4]. Utilizing this band as a 300-kDa marker, the molecular mass of a band extracted from aposymbiotic *P. caudatum* RB-1 cells was calculated to be 266-kDa (lane 2, black arrow). The observation that SAg extracted from the aposymbiotic RB-1 cells manifests as a 266-kDa protein suggests that this particular protein is the SAg labeled by indirect immunofluorescence microscopy in aposymbiotic *P. caudatum* RB-1 cells. However, as shown in Figure 1B,C, SAg bands extracted from *P. tetraurelia* stock 51 (lane 1) and symbiotic *P. caudatum* RB-1 (lane 3) also showed antigenicity against mAb SAgPC, despite the fact that these cells did not show FITC fluorescence via indirect immunofluorescence microscopy. Furthermore, in the extract obtained from the symbiotic RB-1 cells, a 266-kDa band disappeared and two new bands of low molecular masses appeared (lane 3). The molecular masses of these two bands were calculated to be 188 and 149-kDa using pre-stained molecular mass markers (lane 3, red arrows). These 188 and 149-kDa SAgs and 300-kDa SAg of *P. tetraurelia* were labeled by this mAb. These SAg epitopes may have been masked in cells fixed with 4% (*w*/*v*) paraformaldehyde or in living cells and became reactive with the mAb because the mask was removed during the SAg extraction process or SDS-PAGE. Since FITC fluorescence could not be observed in *P. tetraurelia* strain 51 by indirect immunofluorescence, the epitopes of the approximately 300-kDa SAg of this strain might be also masked. Since *Paramecium* clones have the ability to express many different SAgs but each clone expresses only one type of SAg under the same environmental conditions [10], it is an unusual phenomenon for a symbiotic *P. caudatum* clone to simultaneously express two SAgs of different molecular weights.

SAgs are detected in various *Paramecium* species such as *P. caudatum*, *P. multimicronucleatum P. primaurelia*, *P. biaurelia*, *P. triaurelia*, *P. tetraurelia*, *P. octaurelia*, and *P. novaurelia*, [1,15,42,43,44,45]. However, this is the first discovery to demonstrate that *P. caudatum* SAg is replaced with other SAgs if the *P. caudatum* cell is infected by the endonuclear symbiotic bacterium *H. obtusa*. To confirm the species specificity of the cross-reactivities of the mAb SAgPC, 21 *Paramecium* species including *P. caudatum* were examined via indirect immunofluorescence microscopy using cells fixed with 4% (*w*/*v*) paraformaldehyde. All *Paramecium* cells in Table 1 are *Holospora* or *Holospora*-like bacteria (HLB) free cells. The results demonstrated that an epitope capable of reacting with this mAb by indirect immunofluorescence microscopy was detected exclusively in *P. caudatum* RB-1 cells and was not detected in the strains of the other *Paramecium* species examined. However, because the presence of the epitope for mAb SAgPC was confirmed by immunoblotting with cold salt/ethanol extracts of *P. tetraurelia* stock 51 cells, other FITC-negative *Paramecium* species in Table 1 may also show the presence of epitopes via immunoblotting. Table 1 also shows that the mAb binds to the cell surface of aposymbiotic *P. caudatum* RB-1, irrespective of the cell’s phase in the growth cycle, whether in the log phase or the early stationary phase (one day after final feeding at 25 °C).

### 3.2. Immobilization Test of Aposymbiotic and Symbiotic Cells

**Figure 2:**
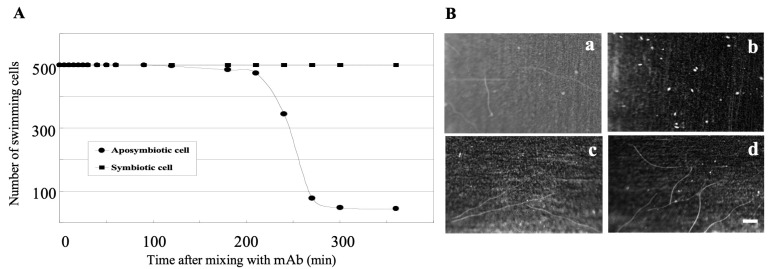
Immobilization test of *P. caudatum* RB-1 cells with mAb SAgPC. (**A**) Kinetics of immobilization test of aposymbiotic and symbiotic RB-1 cells treated with the mAb. Cells in the early stationary phase of growth were mixed with the mAb at 25 °C and observed under a stereomicroscope (see Section 2.2). Note that only the aposymbiotic cells were immobilized, but symbiotic cells were not. Closed circle, aposymbiotic cells. Closed square, symbiotic cell. (**B**) Swimming loci of the aposymbiotic *P. caudatum* RB-1 cells (**a** and **b**) and symbiotic cells (**c** and **d**). Cells were mixed with mAb (**b** and **d**) or with modified Dryl’s solution (MDS) (**a** and **c**) and photographed with 2 s exposures at 270 min after the mixing at 25 °C. Note that only the aposymbiotic cells (**b**) were immobilized by the mAb. To clarify the swimming loci in (**b**–**d**), slight adjustments in brightness and contrast were made in Photoshop without altering or distorting the information in the figure. Scale bar, 1 mm.

It is known that an anti-SAg antibody immobilizes the swimming activity of *Paramecium* cells [8,9,10], so SAg is also called the immobilization antigen (i-antigen). In order to confirm that the mAb SAgPC is able to immobilize the swimming activity of *Paramecium* cells, the aposymbiotic and symbiotic *P. caudatum* RB-1 cells were mixed with the culture medium of the hybridoma cells containing the mAb. The resultant mixture was then observed to determine whether the swimming activity of the cells was immobilized or not. As demonstrated in Figure 2, the aposymbiotic cells exhibited immobilization in response to the mAb, while the symbiotic cells did not. The aposymbiotic cells began to immobilize at 2 h post-mixing with the mAb, and approximately 91% of the cells were immobilized after 5 h (Figure 2A). In contrast, when the symbiotic cells were mixed with MDS instead of the culture medium of the hybridoma cells, both cells remained immobilized (Figure 2B, a and b). These results indicate that the 266-kDa protein is the SAg (=immobilization antigen) whose swimming activity is immobilized by the mAb. However, about 9% of the aposymbiotic cells were not immobilized even 6 h after mixing. These cells might be expressing different SAgs. The epitope of this mAb is present not only in the 266-kDa SAg of aposymbiotic *P. caudatum* but also in 300-kDa SAg of *P. tetraurelia* stock 51 and in the 188- and 149-kDa SAgs of symbiotic *P. caudatum* (Figure 1B,C). However, the epitopes in these cells were not labeled by indirect immunofluorescence microscopy, presumably because these epitopes were masked. Therefore, the epitope of 9% of the aposymbiotic cells in Figure 2A might be masked or the local culture conditions occurring in the test tubes for *Paramecium* culture may have altered the expression of the SAg to a different type of SAg.

### 3.3. Reversibility of SAg Expression

**Figure 3:**
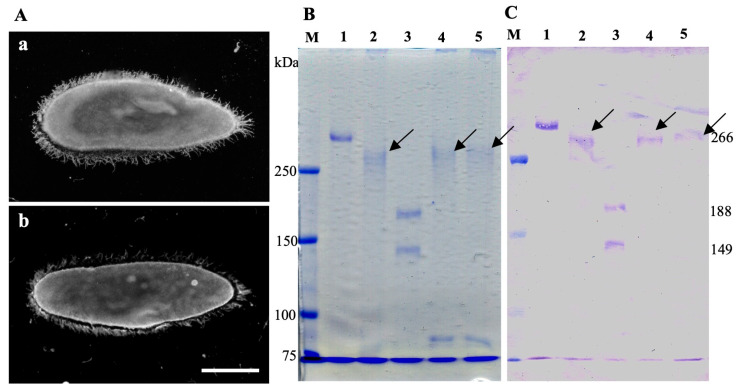
Reversibility of SAg expression in aposymbiotic *P. caudatum* cells obtained from symbiotic cells via penicillin-G-potassium treatment. *Paramecium* cells in the early stationary phase of growth were used. (**A**) Indirect immunofluorescence micrographs with mAb SAgPC. (**a**) Original aposymbiotic *P. caudatum* RB-1 cell before infection with *H. obtusa*. (**b**) Aposymbiotic *P. caudatum* RB-1 cell obtained from symbiotic cells by penicillin treatment. Note that if *H. obtusa* is removed from the macronucleus by penicillin treatment, cell surface of the host *P. caudatum* becomes labeled by indirect immunofluorescence microscopy with the mAb. (**B**) CBB-stained SDS-PAGE gel of cold salt/ethanol extracts. (**C**) Immunoblot of B with the mAb. Lane 1, an extract of *P. tetraurelia* stock 51 cells. Lane 2, an extract of original aposymbiotic *P. caudatum* RB-1 cells. Lane 3, an extract of symbiotic *P. caudatum* RB-1 cells. Lane 4, an extract of original aposymbiotic *P. caudatum* RB-1 cells treated with penicillin. Lane 5, an extract of aposymbiotic cells obtained from penicillin-treated symbiotic cells. Lane M, pre-stained molecular mass markers. Arrow, 266-kDa SAg. Scale bar, 50 μm.

As shown in Figure 1, the symbiotic *P. caudatum* RB-1 cells did not express the 266-kDa SAg. Instead, the cells expressed two new SAgs with low molecular masses of 188 and 149 kDa. To confirm whether changes in SAg expression were truly due to endosymbiosis with *H. obtusa*, bacteria in the host macronucleus were removed from the host cells by penicillin-G-potassium treatment (see Section 2.7), and its effect on SAg expression was examined by indirect immunofluorescence microscopy, SDS-PAGE, and immunoblotting with mAb SAgPC. Three days after the penicillin treatment, several cells were isolated by a micropipette and transferred to test tubes each with a fresh culture medium to establish aposymbiotic clones. The aposymbiotic cells were cultivated for 22 days after the onset of the penicillin treatment and observed by both DIC and indirect immunofluorescence microscopy with anti-*H. obtusa* groEL mAb to confirm the absence of *H. obtusa* in the macronucleus. Immediately after confirming the complete removal of *H. obtusa* from the host cells, indirect immunofluorescence microscopy using mAb SAgPC and the extraction of SAgs via cold salt/ethanol treatment were performed. SAgs were extracted from 2.5 × 10^5^ cells. As shown in Figure 3, the original aposymbiotic *P. caudatum* RB-1 cell (A, a) and the newly established aposymbiotic RB-1 cell (A, b) from the symbiotic RB-1 cell via treatment with penicillin showed FITC fluorescence on their cell surface. Namely, both cells express 266-kDa SAgs on the cell surface. The extract of the original aposymbiotic *P. cautatum* RB-1 treated with penicillin did not show any differences in SAg expression, expressing only 266-kDa SAgs (B and C, lane 4). However, the extract of newly obtained aposymbiotic *P. caudatum* RB-1 cells derived from penicillin-treated symbiotic RB-1 cells recovered the expression of the 266-kDa SAg protein and lost both 188 and 149-kDa bands (B and C, lane 5). These results show that the host cell changes the SAgs reversibly depending on the presence or absence of *H. obtusa* in the macronucleus.

### 3.4. Effects of Starvation and Temperature Shifts on SAg Expression

**Figure 4:**
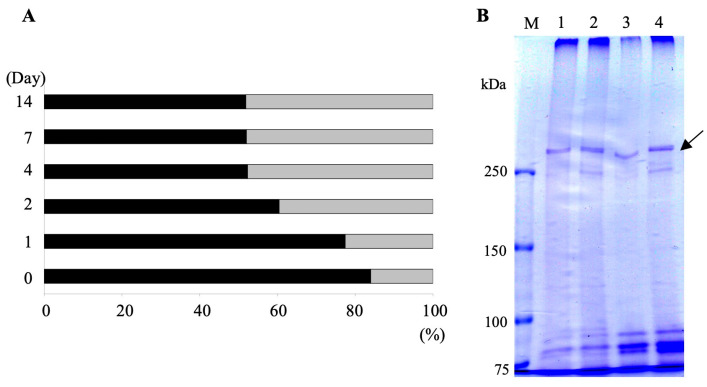
Effects of starvation on 266-kDa SAg expression. (**A**) Aposymbiotic *P. caudatum* strain RB-1 cells in the early stationary phase of growth were washed with MDS and suspended in MDS for 1, 2, 4, 7, and 14 days at 25 °C to induce a state of starvation, and the ratio of cells exhibiting FITC fluorescence on their surfaces was examined via indirect immunofluorescence microscopy using mAb SAgPC by observing 150–200 cells each. Black bars, the percentage of cells showing FITC fluorescence, i.e., the percentage of cells expressing 266-kDa SAg. Gray bars, the percentage of cells showing no FITC fluorescence. (**B**) CBB-stained SDS-PAGE gel. Cell extracts were obtained from starved cells via salt/ethanol extraction 0 (lane 1), 2 (lane 2), 4 (lane 3), and 7 (lane 4) days after washing and loaded onto SDS-PAGE. Slight adjustment in brightness was made in Photoshop without altering or distorting the information in the figure. Lane M, pre-stained molecular mass markers. Arrow, 266-kDa SAg. Note that the 266-kDa band was kept in all lanes and both the 188 and 149-kDa bands did not appear.

The transition from one serotype of SAgs to another has been shown to occur spontaneously at a low frequency or be induced in all cells of the clone simultaneously by modifying the temperature, rate of feeding, antiserum, and other cultural conditions [6,46]. In order to examine whether the change in SAgs is induced by the endosymbiosis with *H. obtusa,* the loss of the 266-kDa SAg, and the appearance of the 188- and 149-kDa SAgs can be induced by other means, we examined the effects of starvation and cultural temperatures on SAg expression. In the early stationary phase, the aposymbiotic *P. caudatum* RB-1 cells were washed, suspended in MDS, and starved. Then, the percent of cells expressing the 266-kDa SAg was counted via indirect immunofluorescence microscopy. As shown in Figure 4A, the ratios of the cells expressing SAg labeled by the mAb 0, 1, 2, 4, 7, and 14 days after the onset of starvation were 84%, 77.4%, 60.4%, 52.3%, 52%, and 51.9%, respectively. This indicates a clear decrease in the ratio of labeled cells, from 84% to 52.3% within just 4 days. Cell extracts obtained from starved cells using the cold salt/ethanol extraction method were loaded onto SDS-PAGE 0, 2, 4, and 7 days after washing the cells. As shown in Figure 4B, the 266-kDa band was present in all lanes, but both the 188- and 149-kDa bands were not induced by starvation. The fact that the 266-kDa band does not fade on SDS-PAGE, even though the percentage of cells showing FITC fluorescence in Figure 4A is almost halved, suggests that the starved cells in (A) may have an increased percentage of SAg with the mAb epitope masked.

**Figure 5:**
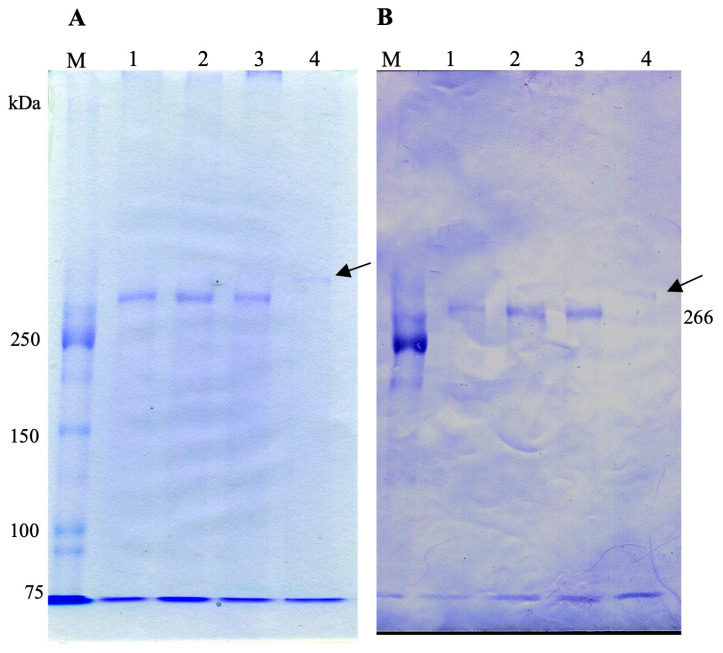
Effect of different temperatures on SAg expression in aposymbiotic *P. caudatum* RB-1 cells. The cells were cultivated at 25 °C, and the cells in the stationary phase of growth were incubated for 24 h at temperatures of 10 °C (lane 1), 15 °C (lane 2), 25 °C (lane 3), and 35 °C (lane 4). Then, their SAgs were extracted by salt/ethanol treatment and loaded onto SDS-PAGE and immunoblot. (**A**) SDS-PAGE gel stained with CBB. (**B**) immunoblot with mAb SAgPC. Note that the molecular mass of SAgs expressed at 10 °C, 15 °C, and 25 °C was 266-kDa. At 35 °C, however, the 266-kDa band disappeared and a new thin SAg band with a slightly higher molecular mass appeared (arrow, lane 4). Lane M, pre-stained molecular mass markers.

Subsequently, the aposymbiotic *P. caudatum* RB-1 cells cultivated at 25 °C and in the stationary phase of growth were subjected to incubation at 10, 15, 25, and 35 °C for 24 h. Thereafter, their SAgs were extracted and loaded onto SDS-PAGE and immunoblots with mAb SAgPC (Figure 5). The 266-kDa SAg band was confirmed at 10, 15, and 25 °C but not at 35 °C. At 35 °C, a new thin SAg band appeared with a molecular mass slightly higher than 266-kDa (arrow, lane 4).

Thus, the 188- and 149-kDa SAgs were not expressed after either starvation or temperature change and, so far, are SAgs whose expression is only induced by endosymbiosis with *H. obtusa*. Since the type of the SAg expressed by the host reversibly changes depending on the absence or presence of *H. obtusa* in the host macronucleus, the 188- and 149-kDa SAgs are predicted to play some essential functions in maintaining endosymbiosis with *H. obtusa* or in suppressing other bacterial infections.

## 4. Discussion

The Gram-negative bacterium *Holospora* and *Holospora*-like bacteria (HLB) (*Alphaproteobacteria, Holosporales*) are endonuclear symbionts of the ciliate *Paramecium* species [47,48,49,50,51,52,53,54,55,56,57,58,59,60,61,62,63,64,65,66,67,68,69,70,71,72,73,74] and the brackish water ciliate *Frontonia salmastra* [75]. Recently, their phylogenetic positions were revised [76,77,78,79]. Since the SAg of *P. caudatum* was reversibly altered by the presence or absence of the endonuclear symbiotic bacterium *H. obtusa*, an understanding of the infection process and behavior of *H. obtusa* in the host macronucleus is necessary to know the timing and function of the change in SAgs.

*H. obtusa* enters the digestive vacuole (DV) through the host cytopharynx, subsequently escaping from the DV into the cytoplasm. Thereafter, it identifies the macronuclear envelope and enters the nucleus. Therefore, it cannot enter the cells in conjugation or early-stage exconjugation, meaning it lacks the ability to form DVs [55,73]. The *Holospora* species are incapable of multiplying outside of the host cell due to their reduced genome size [62,80,81,82] and show species specificity and nucleus specificity in their habitats [63,67,68,69,73,83,84,85,86]. In the case of *H. obtusa*, the macronuclear properties necessary for recognition of the target nucleus and invasion into the nucleus by this bacterium are provided by the examined *P. caudatum*, *P. multimicronucleatum*, and *P. aurelia* species and *P. jenningsi* strains, whereas in *P. bursaria*, *P. putrinum*, *P. duboscqui*, *P. woodruffi*, *P. calkinsi*, *P. polycaryum*, and *P. nephridiatum*, *H. obtusa* is unable to invade the macronucleus, even though *H. otusa* can escape the DV of these *Paramecium* species and emerge in their cytoplasm. Furthermore, stable maintenance of the infected *H. obtusa* in the host macronucleus is achieved only in certain strains of *P. caudatum*. Thus, *H. obtusa* invades the macronucleus of *Paramecium* species closely related to *P. caudatum* but is only maintained in the macronucleus of specific *P. caudatum* strains [63,67,68,69,73,83,84]. The macro- and micronucleus of *P. caudatum* originate from a common fertilization nucleus during the conjugation process. The macronuclear property necessary for it to be recognized and infected by *H. obtusa* is acquired by four of the eight postzygotic nuclei as soon as the four nuclei differentiate morphologically into the macronuclear anlagen [55].

In the infection process of *H. obtusa*, immediately after mixing *H. obtusa* and *P. caudatum* cells, *H. obtusa* cells are engulfed by the host DVs through the cytopharynx. The infectious form (IF, about 13 μm long) of *H. obtusa* differentiates from the activated form (AF) via the acidification of the inside of the DV by asidosomal fusion to the DV, and the AF escapes from the DV into the cytoplasm before lysosomal fusion with the DV. The reproductive short form (RF, about 1.5–2 μm long) and intermediate forms between the RF and IF cannot escape from the DV and are eventually digested by the DVs. The first AF appears in the host macronucleus less than 10 min after mixing with paramecia and begins to differentiate to the RF 32–34 h after invasion into the macronucleus at 25 °C [33,55,63,83]. The RFs propagate by binary fissions in the macronucleus [33,87,88]. When the host cell starves or the host protein synthesis is inhibited with emetine, a eukaryotic cell-specific protein synthesis inhibitor, the RFs stop dividing and differentiate into the IFs via the intermediate forms [89]. About half of the length of this IF consists of a cytoplasmic region with two nucleoids stained with 4′,6-diamidino-2-phenylindole dihydrochloride (DAPI) [33,90]. The other half consists of a periplasmic region involving a special tip called an invasion tip [38,87,90,91]. The cytoplasmic regions, periplasmic region, and invasion tip of the IF can be readily identified by a phase-contrast or differential-interference-contrast microscope (DIC) [90,91]. However, unlike IF, RF does not show such subcellular differentiation, and DAPI fluorescence is distributed throughout the cell [33,63,65,67,68,69,73,88,90].

The *H. obtusa*-bearing symbiotic *P. caudatum* cells express a high level of heat shock protein genes *hsp60* and *hsp70*. This allows host cells to exhibit high viability when transferred from the normal culture temperature of 25 °C to the unsuitable growth temperature of 35 °C [31]. The *H. obtusa*-free aposymbiotic *P. caudatum* cells almost cease swimming at both 4 °C and 40 °C, although cells with *H. obtusa* can swim at these temperatures [92]. *Paramecium* cells bearing the micronucleus-specific *H. elegans* also express high levels of *hsp70* mRNA, even at 25 °C, and the host cells acquire heat-shock resistance and survive better than aposymbiotic paramecia, even at 37 °C [32]. Curiously, this overexpression of the *hsp70* gene of the host cells continues irreversibly, even after the removal of *H. elegans* by treatment with penicillin [32]. Nakamura et al. [30] identified six genes of *P. caudatum*, in addition to *hsp60* and *hsp70*, which were differentially expressed in aposymbiotic and symbiotic cells using differential-display reverse-transcribed PCR. Furthermore, *Holospora*-bearing *Paramecium* acquires osmotic stress resistance [93,94] and various metal chloride resistance [95]. This evidence shows that infection with the *Holospora* species alters the host gene expression.

The present results suggest that *H. obtusa* in the macronucleus induces the expression of specific SAgs by altering gene expression in the host. Within 20 days of the onset of endosymbiosis with *H. obtusa*, this SAg was replaced with low-molecular-mass 188 and 149-kDa SAgs. Furthermore, when *H. obtusa* cells in the macronucleus were completely removed by penicillin, within 22 days of the penicillin treatment, the resulting aposymbiotic cells recovered the 266-kDa SAg again and lost the 188 and 149-kDa SAgs. Therefore, these two SAgs may be involved in maintaining only the *Holospora* species that are currently in endosymbiosis with the host cell or maintaining only *H. obtusa* and preventing dual infection by other *Holospora* species. However, the invasion of *H. obtusa* from the host DVs to the host macronucleus occurs within 10 min of mixing them, and the IFs differentiate the RFs 32–34 h after mixing at 25 °C [33,55,63,83]. *P. caudatum* cells that lost the infected *H. obtusa* from the macronucleus appear 3 days after the penicillin treatment. Therefore, to determine the relationship between the timing of the appearance and disappearance of *H. obtusa* in the host macronucleus and the timing of the changes in the host SAgs, it is necessary to examine the changes in SAg over a much shorter period.

How does *H. obtusa* alter the host SAg gene expression after invading the macronucleus of the host *P. caudatum*? It is known that the OspF effector protein of *Shigella flexneri* regulates the host gene expression via the modification of the host nuclear protein [96]. Furthermore, it is also known that the AnkA effector protein of the pathogen *Anaplasma phagocytophilum* is secreted and binds to the host DNA [97]. The AnkA interacts with transcriptional regulatory regions of the *CYBB* locus at sites where transcriptional regulators bind [98]. Although the mechanism by which *H. obtusa* alters the host gene expression remains to be elucidated, it has recently been confirmed that 3 h after the invasion of *H. obtusa* IFs into the host macronucleus, not only the pre-existing 63-kDa protein (periplasmic region protein 1, PRP1) in the large periplasmic region of the IF but also the newly synthesized PRP1 proteins after invasion into the macronucleus are also secreted into the host macronucleus and remain in the nucleus [99]. This PRP1 protein is IF-specific and one of the most abundant proteins of the IF. In addition, the PRP1 protein had a putative signal peptide comprising 24 amino acids near the C-terminal of the protein. Furthermore, a Pfam motif search on Genome Net (https://www.genome.jp/, accessed on 28 November 2022) showed that the PRP1 protein had a DNA-binding domain, D5_N at aa369–491, and had been confirmed to bind with macronuclear DNA [100]. Thus, this PRP1 protein is the most likely candidate predicted to induce changes in the host gene expression, not only for the *hsp60* and *hsp70* genes but also for the SAg gene. Therefore, it is essential to clarify whether the timing of the replacement of the 266-kDa SAg by the 188 and 149-kDa SAgs after mixing *P. caudatum* and *H. obtusa* and the timing of PRP1 secretion into the macronucleus are the same, which will be revealed in future studies.

What is the function of the 188 and 149-kDa SAgs in the symbiotic cells? Many SAgs, known as GPI-anchored proteins in other organisms, function as receptors for signal transduction. Furthermore, it is known that the GPI-anchored protein interacts with the src-family kinase [101]. The symbiotic-specific SAgs may be concerned with the exogenous signal response needed for the endosymbiosis of *H. obtusa*. Therefore, the function of the SAg in endosymbiosis will be clarified by findings of exogenous signals that affect the selective expression of the SAg. Starvation and various temperatures could not induce the expression of the 188 or 149-kDa SAgs in the aposymbiotic cell. Various ion strengths also did not induce these two SAgs to the aposymbiotic cell (M. Fujishima, unpublished observation). To date, the expression of the 188- and 149-kDa SAgs has been observed to be induced exclusively under conditions of endosymbiosis with *H. obtusa*. Fujishima and Fujita [83] showed that IFs of *H. obtusa* infected all *P. caudatum* strains examined, differentiated into the RFs, and began to multiply via binary fissions in the macronucleus. However, in about 50% of the *P. cuadatum* strains, *H. obtusa* disappeared from their macronucleus within two weeks of infection. Namely, only certain strains of *P. caudatum* can maintain *H. obtusa* while others cannot. Furthermore, no strains have yet been found to maintain *H. obtusa* belonging to syngen 12. The strain-specific disappearance of *Holospora* from the host nucleus after infection has also been reported for *H. accuminata* in *Paramecium bursaria* [102]. Therefore, if *P. caudatum* strains that cannot maintain *H. obtusa* infecting the macronucleus fail to express 188 and 149-kDa SAgs, it may be possible to show that the 188 and 149-kDa SAgs play an essential role in maintaining *H. obtusa*. This needs to be confirmed in the future.

On the other hand, *H. obtusa* is a macronucleus-specific symbiont of *P. caudatum*, whereas *H. elegans*, *H. undulata*, and *H. recta* are all micronucleus-specific symbionts of *P. caudatum*. Therefore, it seems that *H. obtusa* can coexist with the alternative micronucleus-specific one in the same cell. However, there are no confirmed cases of coexistence of these *Holospora* in the same host cell in nature. The stable coexistence of micronucleus-specific *Holospora* of different species in the same host cell has also not been observed. Furthermore, when *H. elegans* was mixed with the external fluid of *P. caudatum* with *H. obtusa* in the macronucleus, the *H. elegans* could infect the micronucleus of the cell but disappeared from the micronucleus without proliferating. On the other hand, when *P. caudatum* with *H. elegans* in the micronucleus was given as *H. obtusa*, *H. obtusa* could infect the macronucleus but also disappeared immediately from the macronucleus (M. Fujishima, unpublished observation). It is not yet clear how these two *Holospora* species interfere with each other, even though they are separately present in different nuclei of the host cell.

The following phenomena require further investigation and have been identified as potential areas for future research: (1) What is the subcellular localization of the 188- and 149-kDa SAgs in symbiotic cells? (2) When and how does *H. obtusa* alter host SAg gene expression during the infection process? (3) What are the functions of the 188- and 149-kDa SAgs in symbiotic cells?

## Figures and Tables

**Table 1:** Cross-reactivity of mAb SAgPC to *Paramecium* species obtained via indirect immunofluorescence microscopy.

*Paramecium* Species	Strains	Cross-Reactivity	
		Log Phase	Stationary Phase
*P. primaurelia*	HV15-1	−	−
*P. biaurelia*	537	−	−
*P. triaurelia*	136	−	−
*P. tetraurelia*	Stock 51	−	−
*P. pentaurelia*	87	−	−
*P. sexaurelia*	GSZ-3	−	−
*P. septaurelia*	325	−	−
*P. octaurelia*	137	−	−
*P. novaurelia*	91YB1-3	−	−
*P. decaurelia*	223	−	−
*P. undecaurelia*	219	−	−
*P. dodecaurelia*	246	−	−
*P. tredecaurelia*	321	−	−
*P. quqdecaurelia*	328	−	−
*P. polycarium*	YnA(+)	−	−
*P. jenningsi*	30997	−	−
*P. dubosqui*	702	−	−
*P. trichium*	OM4	−	−
*P. calkinsi*	GN5-3	−	−
*P. multimicronucleatum*	TH103	−	−
*P. caudatum*	RB-1	+	+

*Paramecium* species in log phase and stationary phase of growth fixed with 4% (*w*/*v*) paraformaldehyde and examined cross-reactivity of mAb SAgPC by indirect immunofluorescence microscopy. FITC fluorescence was detected only in *P. caudatum* RB-1 cells but not in strains of other *Paramecium* species examined. −, absence of FITC fluorescence. +, presence of FITC fluorescence.

## Data Availability

The original contributions presented in this study are included in the article. Further inquiries can be directed to the corresponding author.
